# Analysis of preoperative influential factors and construction of a predictive nomogram of difficult thyroidectomy

**DOI:** 10.1186/s12893-023-01990-z

**Published:** 2023-04-17

**Authors:** Meng Dong, Jun-Long Song, Lin-Lin Hu, Chen-Chen Hong, Xin-Yue Nie, Zhong Wang, Shi-Chong Liao, Feng Yao

**Affiliations:** Department of Breast and Thyroid Surgery, Renmin Hospital of Wuhan University, Wuhan, 430060 China

**Keywords:** Difficult thyroidectomy, Nomogram, Predictive factors, Thyroid surgery

## Abstract

**Objective:**

To explore the preoperative influential factors of difficult thyroidectomy and establish a preoperative nomogram for predicting the difficulty of thyroidectomy.

**Methods:**

A total of 753 patients who underwent total thyroidectomy with central lymph node dissection between January 2018 and December 2021 were retrospectively enrolled in this study and randomly divided into training and validation groups at a ratio of 8:2. In both subgroups, the patients were divided into difficult thyroidectomy and nondifficult thyroidectomy groups based on the operation time. Patient age, sex, body mass index (BMI), thyroid ultrasound, thyroid function, preoperative fine needle aspiration (FNA), postoperative complications and other data were collected. Logistic regression analysis was performed to identify the predictors of difficult thyroidectomy, and a nomogram predicting surgical difficulty was created.

**Results:**

Multivariate logistic regression analysis demonstrated that male sex (OR = 2.138, 95% CI 1.055–4.336, *p* = 0.035), age (OR = 0.954, 95% CI 0.932–0.976, *p* < 0.001), BMI (OR = 1.233, 95% CI 1.106–1.375, *p* < 0.001), thyroid volume (OR = 1.177, 95% CI 1.104–1.254, *p* < 0.001) and TPO-Ab (OR = 1.001, 95% CI 1.001–1.002, *p* = 0.001) were independent risk factors for difficult thyroidectomy. The nomogram model incorporating the above predictors performed well in both the training and validation sets. A higher postoperative complication rate was found in the difficult thyroidectomy group than in the nondifficult thyroidectomy group.

**Conclusion:**

This study identified independent risk factors for difficult thyroidectomy and created a predictive nomogram for difficult thyroidectomy. This nomogram may help to objectively and individually predict surgical difficulty before surgery and provide optimal treatment.

## Introduction

Thyroid cancer is the most frequently occurring endocrine malignancy. The number of new cases of global thyroid cancer in 2020 reached 586,000, ranking 9th in global tumor incidence [1]. As the main treatment for thyroid cancer, thyroidectomy is becoming widely used in the clinic. With the improvement of surgical technology and the understanding of thyroid anatomy and physiology, thyroidectomy has now become a safer operation [2]. However, thyroidectomy is still difficult because the thyroid gland is rich in blood supply and is adjacent to parathyroid glands and important nerves. In addition, a series of complications, such as bleeding, hypoparathyroidism, recurrent laryngeal nerve (RLN) injury, and chyle leakage, will also seriously affect the quality of survival in patients [3]. Difficult thyroidectomies are often characterized by longer operative time, more intraoperative bleeding, and a higher incidence of postoperative complications [4]. The literature shows that difficult thyroidectomy (DT) is related to goiter, inflammation, hyperthyroidism and other factors [4–8]. However, the degrees of thyroidectomy difficulty caused by various factors are different and difficult to predict. Surgical difficulty is an urgent concern for surgeons, as it is closely related to the effect and safety of thyroidectomy. Therefore, effective and objective approaches are urgently required to determine preoperative factors in thyroidectomy and evaluate the difficulty preoperatively.

## Methods

### Study population

**Fig. 1:**
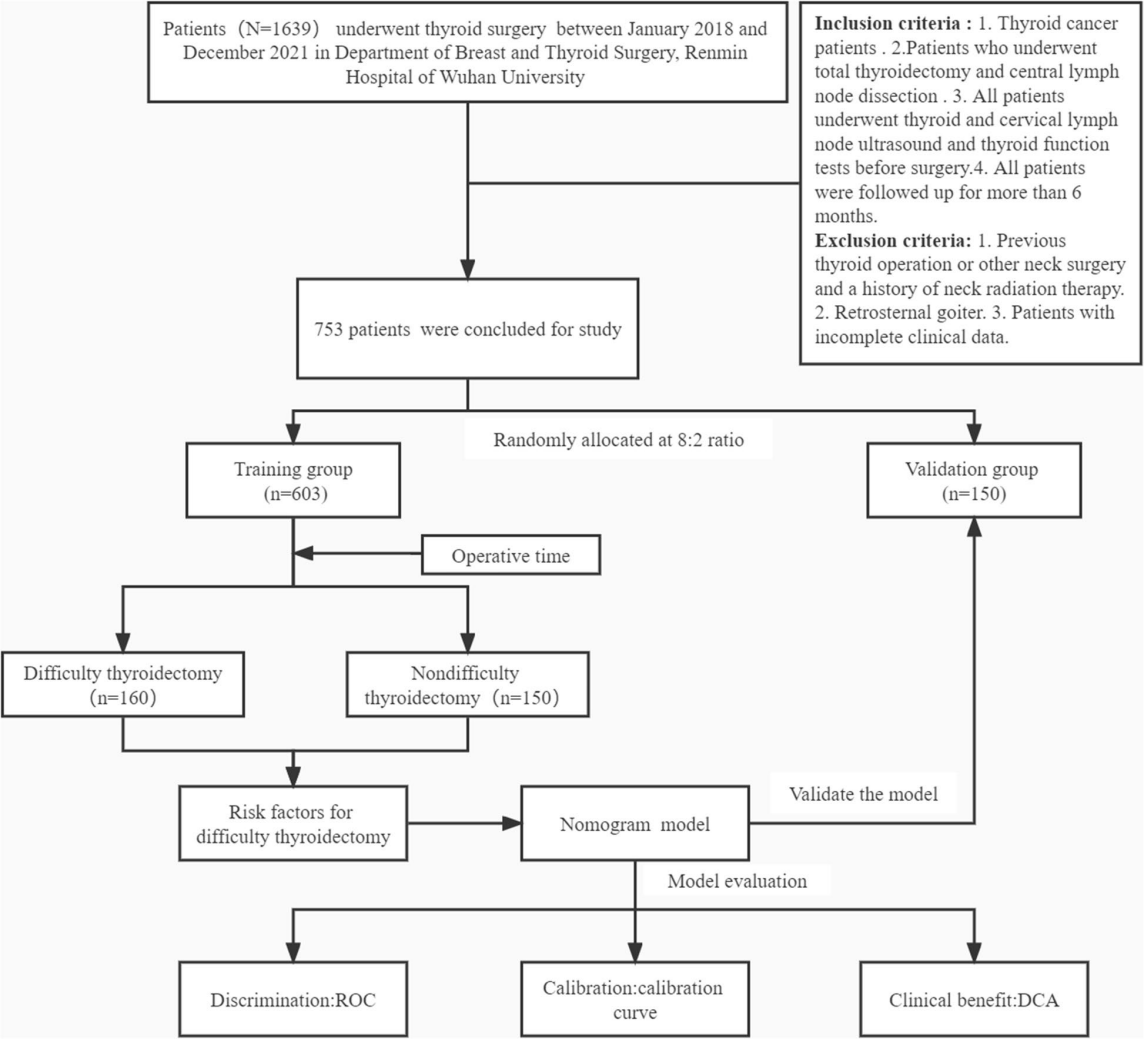
Detailed process. Abbreviations: DCA, decision curve analysis; ROC, receiver operating characteristic curve

All patients (*N* = 1639) who underwent thyroidectomy performed by the same three experienced thyroid surgeons between January 2018 and December 2021 in the Department of Breast and Thyroid Surgery, Renmin Hospital of Wuhan University, were screened for possible inclusion in the study. The inclusion criteria were as follows: 1. Pathologically confirmed diagnosis of thyroid cancer. 2. Patients who underwent total thyroidectomy and central lymph node dissection (CLND). 3. Patients were followed up for more than 6 months. The exclusion criteria were as follows: 1. Previous neck surgery or radiation therapy history 2. Retrosternal goiter 3. Patients with incomplete data. Ultimately, 753 patients were selected and randomly divided into the training and validation groups at a ratio of 8:2. The study was conducted in accordance with the Declaration of Helsinki (as revised in 2013). All patients signed informed consent. The procedure is shown in Fig. 1.

### Data collection

The clinical data included age, sex, BMI, thyroid ultrasound data, such as the volume and blood flow of the thyroid, the size and location of the dominant nodule, nodule close to the capsule and unifocal or multifocal lesions; free triiodothyronine (FT3), free thyroxine (FT4), thyroid-stimulating hormone (TSH), thyroglobulin antibody (Tg-Ab, normal range: 0–60 U/ml), and thyroid peroxidase antibody (TPO-Ab, normal range: 0–60 U/ml); preoperative FNA; preoperative and postoperative vocal cord assessment by laryngoscopy; operative time; and postoperative complications.

### Operative procedures

Under general endotracheal anesthesia, all patients underwent open total thyroidectomy with CLND. A 5–6 cm transverse collar skin incision was made into the midline of the anterior neck 2 cm above the sternal notch. The skin and subcutaneous tissues were separated, and subplatysmal flap dissection was carried out inferiorly from the sternal notch to the hyoid bone after exposing the lower layer of the platysma. Then, the midline of the strap muscle was divided to expose the thyroid gland. Dissection of the total thyroid and central lymph node was performed, and the parathyroid glands and RLN were preserved. A drainage tube was placed, and the incision was sutured. Bipolar coagulation forceps were used to stop bleeding in all operations.

### Difficult thyroidectomy

The operative time was defined as the time from skin incision to wound closure and was obtained from the anesthetic record sheet. It was taken as a numerical measure of thyroidectomy difficulty. With the same operator and surgical setting, a thyroidectomy with a longer operative time was defined as a DT. The patients were ordered by operative time. Those with an operative time above the 75th percentile were grouped as difficult thyroidectomy (DT), while those with an operative time below the 25th percentile were considered nondifficult thyroidectomy (NDT).

### Outcomes

The main outcomes were surgical wound infection, surgical reintervention for postoperative hemorrhage, and RLN injury (paralysis of the vocal cord determined by postoperative laryngoscopy and/or symptoms of hoarseness and was considered permanent if it persisted for more than 6 months). and hypoparathyroidism. Temporary hypoparathyroidism was defined as a postoperative serum calcium level less than 2.00 mmol/L. Permanent hypoparathyroidism was defined as a continuous need for calcium and/or vitamin D supplements to maintain normal calcium levels at six months or more after surgery.

### Statistical analysis

SPSS 25.0 and R software (version 4.2.0) were used for the statistical analysis. The Kolmogorov‒Smirnov test was used to test the normality of the distribution of continuous variables. Normally distributed data are presented as the means and standard deviations (‾x ± s) and were compared by the T test. Nonnormally distributed data are presented as medians and interquartile ranges [M (Q1, Q3)] and were analyzed using the Mann‒Whitney U test. Categorical data are expressed as counts and percentages [n (%)], and the chi-square test, Fisher’s exact test, or Pearson’s correlation analysis were applied. Independent risk factors for difficult thyroidectomy were analyzed by univariate and multivariate logistic regressions. Differences of *p* < 0.05 were considered statistically significant.

### Construction and verification of the predictive model

Using R software, a clinical prediction nomogram was constructed based on the results of the final multivariable logistic regression. To quantify the predictive ability of the nomogram, the area under the curve (AUC) of the receiver operating characteristic curve (ROC), the calibration curve and decision curve analysis (DCA) were conducted.

## Results

### Clinical characteristics

A total of 753 patients were enrolled in the study, among which 603 patients were randomized to the training group (160 in DT, 150 in NDT) and 150 to the validation group (40 in DT, 42 in NDT). The detailed demographics and clinical characteristics of the patients are given in Table 1.

**Table 1:** Patient characteristics in the training and validation groups

	Training group *n* = 603	Validation group *n* = 150	*p*
Operative time(min)	120(99, 135)	120(95,138.5)	0.694
Sex			0.532
male	139(23.1%)	31(20.7%)	
female	464(76.9%)	119(79.3%)	
Age(y)	48(37,55)	50(39,56)	0.157
BMI (kg/cm^2^)	23.72 ± 3.34	23.40 ± 3.47	0.162
Thyroid volume(mL)	11.02(8.92,14.65)	10.38(8.27,14.04)	0.145
Blood flow of thyroid			0.423
rich	169(28.0%)	47(31.3%)	
normal	434(72.0%)	103(68.7%)	
Tumor size(cm)	0.88(0.57,1.40)	0.90(0.66,1.30)	0.420
Tumor location			0.687
upper	102(16.9%)	23(15.3%)	
middle	322(53.4%)	86(57.3%)	
lower	179(29.7%)	41(27.4%)	
Close to the capsule			0.257
yes	264(43.6%)	58(38.7%)	
no	339(56.4%)	92(61.3%)	
Lesion			0.656
unifocal	158(26.2%)	42(28.0%)	
multifocal	445(73.8%)	108(72.0%)	
FNA			0.283
yes	246(40.8%)	54(36.0%)	
no	357(59.2%)	96(64.0%)	
FT3(pg/mL)	3.35(3.10,3.62)	3.23(3.02,3.55)	0.016
FT4(ng/dL)	1.17(1.07,1.29)	1.19(1.06,1.30)	0.374
TSH(uIU/mL)	1.67(1.16,2.41)	1.69(1.08,2.30)	0.231
Tg-Ab(U/mL)	20.40(15.00,73.25)	22.10(15.00,54.43)	0.674
TPO-Ab(U/mL)	38.50(28.00,57.40)	42.70(30.80,116.93)	0.067

*Abbreviations*: *BMI* body mass index, *FT3* free triiodothyronine, *FT4* free thyroxine, *TSH* thyroid-stimulating hormone, *Tg-Ab* thyroglobulin antibody, *TPO-Ab* thyroid peroxidase antibody

### A comparison of the temporary postoperative complications in DT and NDT

A total of 98 (25.0%) complications were encountered in the DT (69, 17.6%) and NDT (29,7.4%) groups. Of these 98 cases, 4 (1.0%) experienced surgical reintervention for postoperative hemorrhage, 3 (0.8%) had surgical wound infections, 19 (4.8%) had transient RLN injuries, 3 (0.8%) had permanent RLN injuries, 67 (17.1%) experienced transient hypoparathyroidism, and 2 (0.5%) had permanent hypoparathyroidism. The results showed that the incidence rates of transient hypoparathyroidism (22.5% vs. 11.5%, *p* = 0.004) and transient RLN injury (7.0% vs. 2.6%, *p* = 0.043) were higher in the DT group than in the NDT group. However, no significant differences were observed in the incidences of surgical wound infection (1.5%vs. 0%, *p* = 0.261), postoperative hemorrhage (1.5% vs. 0.5%, *p* = 0.644), permanent hypoparathyroidism (1% vs. 0%, *p* = 0.496) and permanent RLN injury (1% vs. 0.5%, *p* = 0.586) between the two groups (Table 2).

**Table 2:** Comparisons of the operative complications in the DT and NDT groups

Operative complications	DT and NDT in two groups *n* = 392	DT in two groups *n* = 200	NDT in two groups *n* = 192	*p*
Complications	98(25.0%)	69(34.5%)	29(15.1%)	< 0.001
Postoperative hemorrhage	4(1.0%)	3(1.5%)	1(0.5%)	0.64
Surgical wound infection	3(0.8%)	3(1.5%)	0(0%)	0.26
RLN injury				
Transient	19(4.8%)	14(7.0%)	5(2.6%)	0.04
Permanent	3(0.8%)	2(1.0%)	1(0.5%)	0.59
Hypoparathyroidism				
Transient	67(17.1%)	45(22.5%)	22(11.5%)	0.004
Permanent	2(0.5%)	2(1.0%)	0(0%)	0.50

*Abbreviations*: *DT* difficult thyroidectomy, *NDT* nondifficult thyroidectomy, *RLN* recurrent laryngeal nerve

### Analysis of risk factors

The univariate analysis showed that male sex (*p* = 0.001), age (*p* < 0.001), BMI (*p* < 0.001), thyroid volume (*p* < 0.001), Tg-Ab (*p* = 0.003) and TPO-Ab (*p* = 0.001) were associated with DT (Table 3). Multivariate logistic regression analyses were conducted to analyze the variables showing statistically significant differences in the univariate analysis. The results showed that male sex (OR = 2.138, 95% CI 1.055–4.336, *p* = 0.035), age (OR = 0.954, 95% CI 0.932–0.976, *p* < 0.001), BMI (OR = 1.233, 95% CI 1.106–1.375, *p* < 0.001), thyroid volume (OR = 1.177, 95% CI 1.104–1.254, *p* < 0.001), and TPO-Ab (OR = 1.001, 95% CI 1.001–1.002, *p* = 0.001) were independent predictors for DT (Table 4).

**Table 3:** Univariate analysis of risk factors for DT in the training group

	DT *n* = 160	NDT *n* = 150	*p*
Sex			0.001
male	47(29.4%)	20(13.3%)	
female	113(70.6%)	130(86.7%)	
Age(y)	45(32,52)	50(39,57.25)	< 0.001
BMI (kg/cm^2^)	24.55 ± 3.54	22.61 ± 2.58	< 0.001
Thyroid volume(mL)	13.19(9.59,19.01)	10.04(8.09,11.64)	< 0.001
Blood flow of thyroid			0.213
rich	36(22.5%)	43(28.7%)	
normal	124(77.5%)	107(71.3%)	
Tumor size(cm)	0.95(0.59,1.70)	0.85(0.59,1.53)	0.732
Tumor location			0.321
upper	30(18.8%)	25(16.7%)	
middle	95(59.4%)	81(54.0%)	
lower	35(21.8%)	44(29.3%)	
Close to the capsule			0.953
yes	72(45.0%)	68(45.3%)	
no	88(55.0%)	82(54.7%)	
Lesion			0.425
unifocal	47(29.4%)	38 (25.3%)	
multifocal	113(70.6%)	112(74.7%)	
FNA			0.200
yes	69(43.1%)	54(36.0%)	
no	91(56.9%)	96(64.0%)	
FT3(pg/mL)	3.44(3.19,3.68)	3.35(3.10,3.67)	0.214
FT4 (ng/dL)	1.15(1.06,1.30)	1.18(1.07,1.28)	0.866
TSH(μIU/mL)	1.58(1.06,2.38)	1.61(1.14,2.29)	0.922
Tg-Ab(U/mL)	20.45(15.00,133.13)	20.35(10.10,34.65)	0.003
TPO-Ab(U/mL)	40.85(28.23,1135.98)	36.10(25.28,51.30)	0.001

*Abbreviations*: *BMI* body mass index, *DT* difficult thyroidectomy, *FT3* free triiodothyronine, *FT4* free thyroxine, *NDT* nondifficult thyroidectomy, *Tg-Ab* thyroglobulin antibody, *TPO-Ab* thyroid peroxidase antibody, *TSH* thyroid-stimulating hormone

**Table 4:** Multivariate logistic regression analysis of risk factors for DT in the training group

	β	*p*	OR (95% CI)
Male sex	0.760	0.035	2.138(1.055-4.336)
Age(years)	-0.047	< 0.001	0.954(0.932-0.976)
BMI (kg/cm^2^)	0.210	< 0.001	1.233(1.106-1.375)
Thyroid volume(mL)	0.163	< 0.001	1.177(1.104-1.254)
Tg-Ab(U/mL)	0.002	0.123	1.002(1.000-1.003)
TPO-Ab(U/mL)	0.001	0.001	1.001(1.000-1.002)

*Abbreviations*: *CI* confidence interval, *Tg-Ab* thyroglobulin antibody, *TPO-Ab* thyroid peroxidase antibody

### Construction of the predictive model

**Fig. 2:**
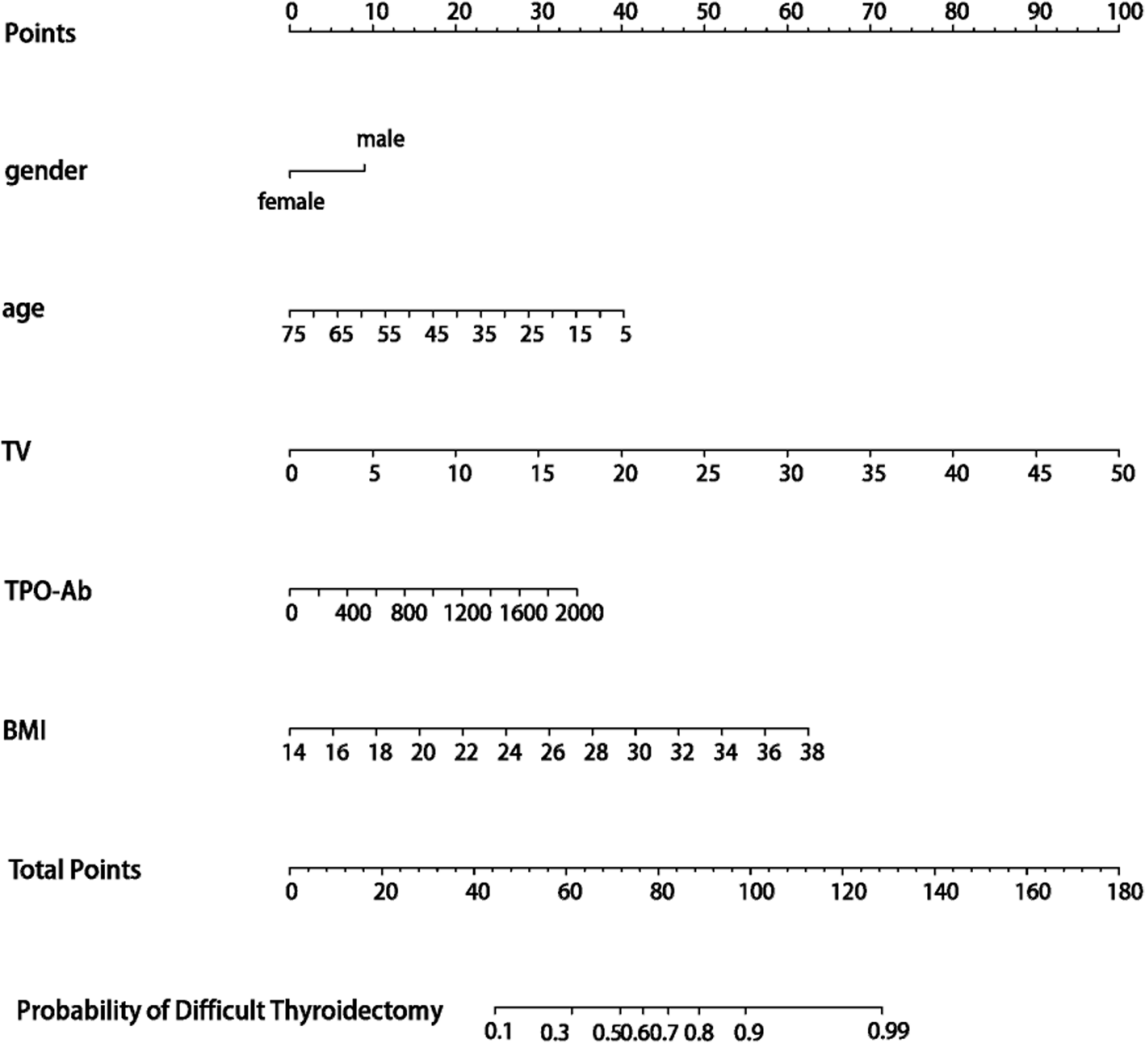
The nomogram for predicting the difficulty of thyroidectomy preoperatively. Abbreviations: TV, thyroid volume; TPO-Ab, thyroid peroxidase antibody; BMI, body mass index

Based on the independent risk factors shown in Table 4, the visualized nomogram predicting the risk of DT was constructed in R software and is shown in Fig. 2. Using the nomogram, each prediction variable was scored, and the sum of the scores was obtained as the total score. The predicted risk corresponding to this total score was the probability of DT.

### Verification of the predictive model

**Fig. 3:**
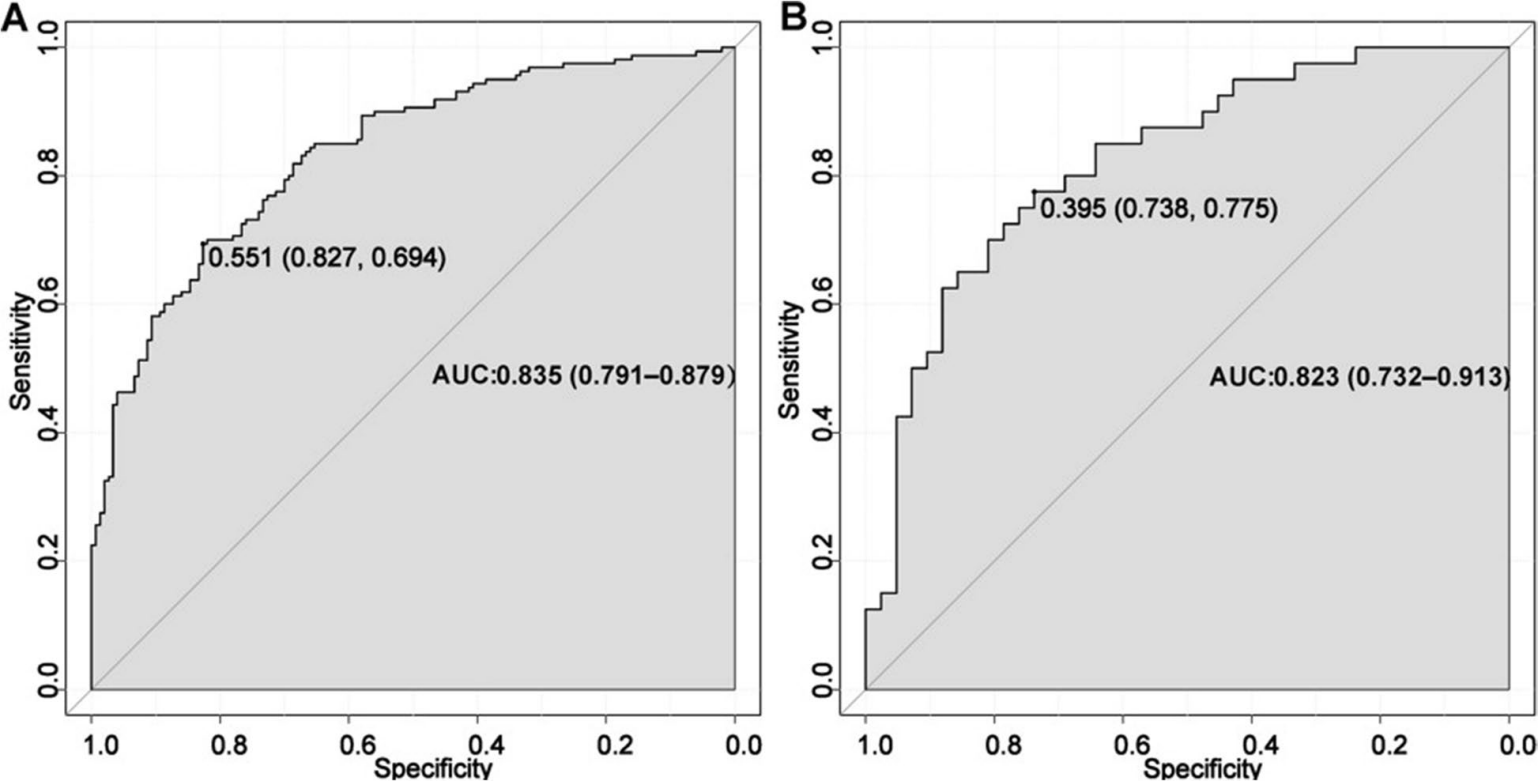
The AUCs of the training group and validation group

**Fig. 4:**
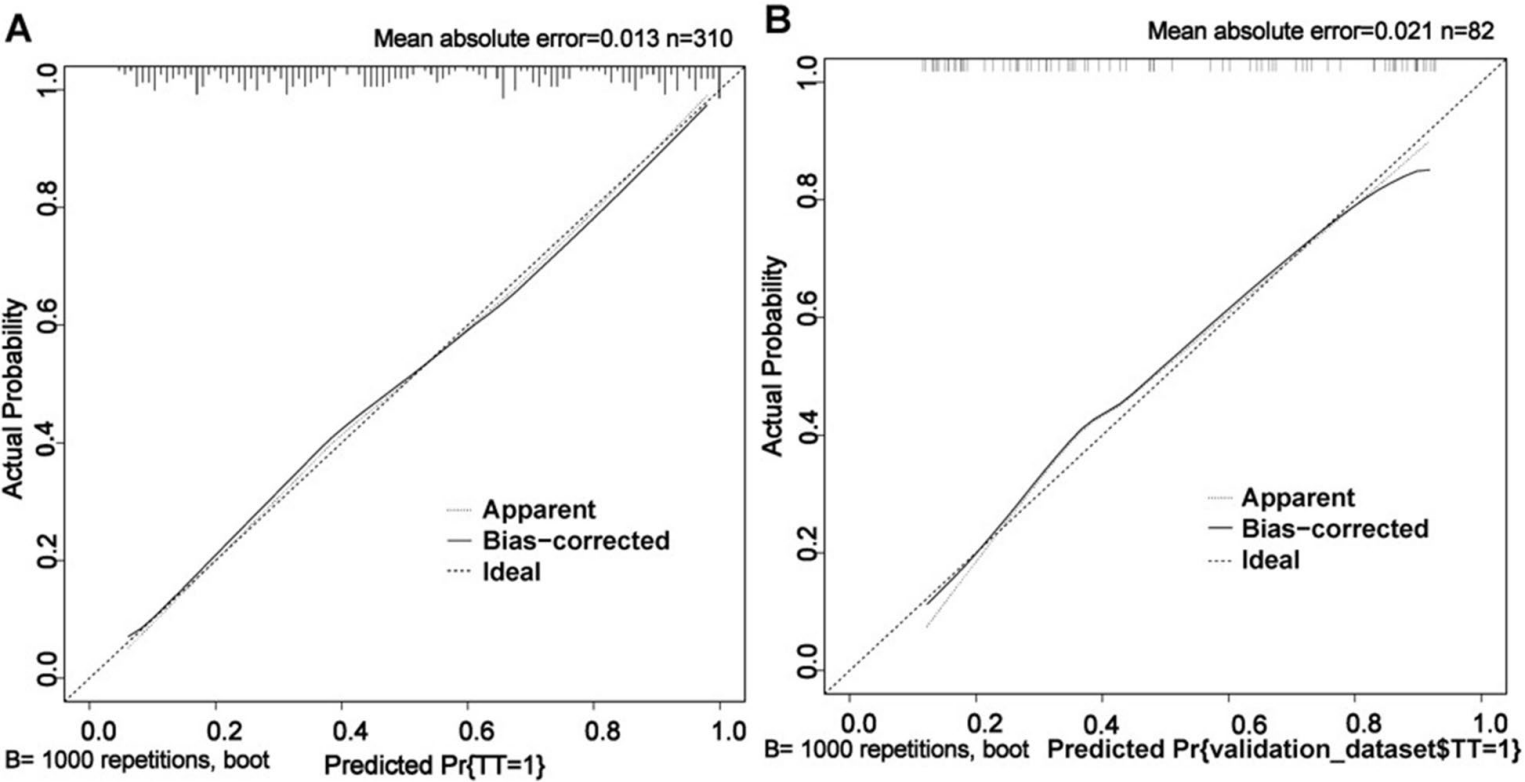
The calibration curves of the training group and validation group

**Fig. 5:**
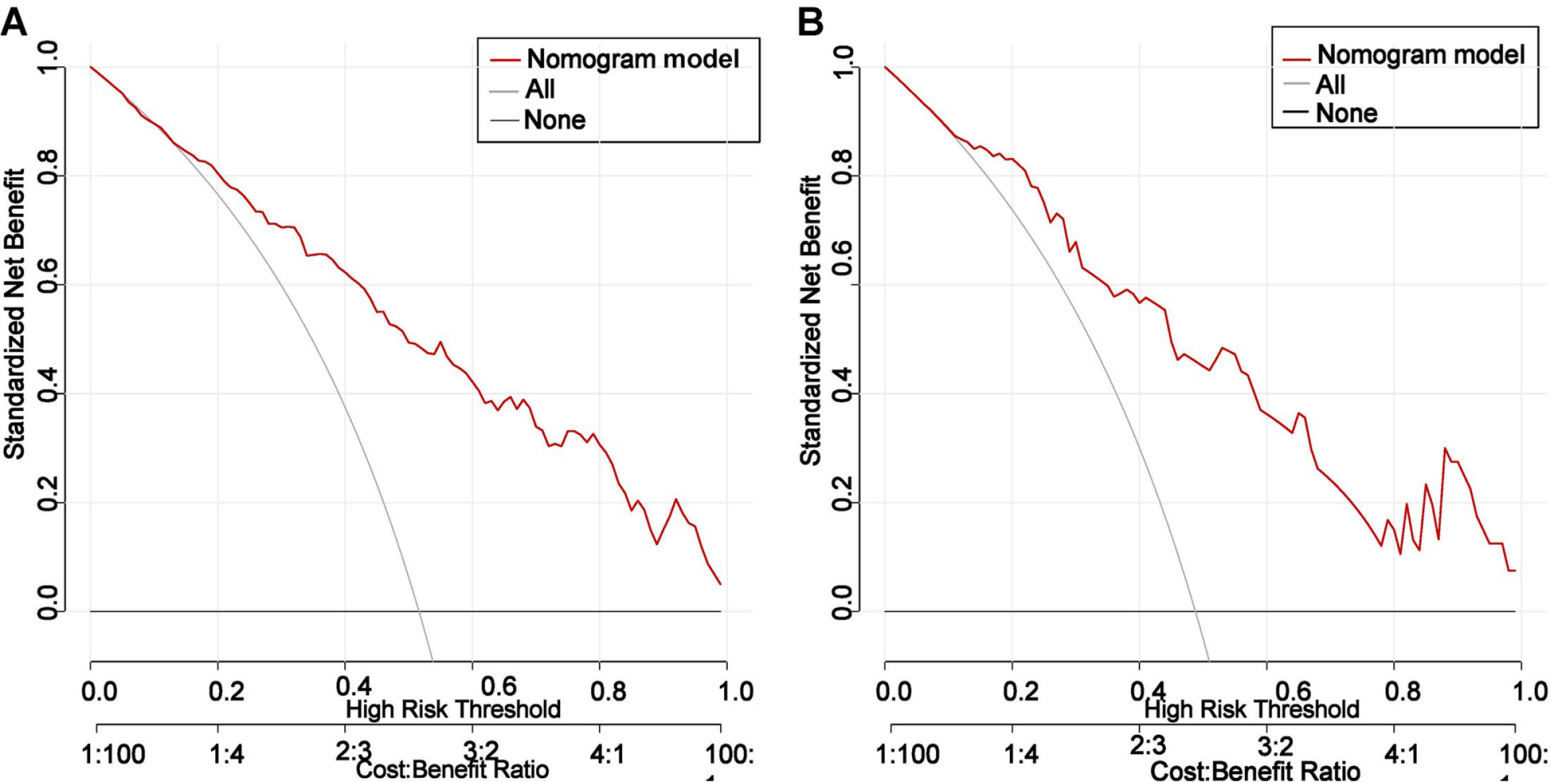
DCA of the nomogram in the training and validation groups

The AUCs (Fig. 3) for the training group and validation group were 0.835 (95% CI: 0.791–0.879) and 0.823 (95% CI: 0.732–0.913), respectively, indicating the favorable discrimination and prediction capabilities of the nomogram. The calibration curves (Fig. 4) showed that the predicted curves were close to the ideal curves, demonstrating high agreement between the nomogram predictions and actual outcomes. DCA (Fig. 5) was established to assess the net benefit of the nomogram to the decision and showed that using the nomogram can lead to more clinical benefits.

The ROCs of the nomogram in the training (Fig. 3A) and validation (Fig. 3B) cohorts. The nomogram had good discriminative performance, with areas under the ROC curve (AUC) (95% confidence interval) of 0.835 (95% CI: 0.791–0.879) and 0.823 (95% CI: 0.732–0.913) in the training and validation cohorts, respectively.

Calibration curves of the nomogram in the training (Fig. 4A) and validation (Fig. 4B) cohorts (bootstrap 1000 repetitions). The nomogram-predicted probability of difficult thyroidectomy is plotted on the x-axis, and the actual probability is plotted on the y-axis. The 45-degree line represents a perfect prediction by an ideal model, and the solid line represents the performance of our nomogram, which lays closer to the ideal line, indicating a good performance of the nomogram.

Decision curve analysis (DCA) of the nomogram in the training (Fig. 5A) and validation (Fig. 5B) cohorts. The gray solid line represents the difficult operation risk nomogram; the black dotted line represents the assumption that all patients are difficult surgery; and the solid black line represents the assumption that no patients are difficult surgery. The decision curve revealed that using the nomogram to predict the difficulty of thyroidectomy adds more net benefit than any of the other predictors alone.

## Discussion

With the increasing incidence of thyroid cancer, there has been growing interest in risk factors for DT, as surgery is the most important treatment. Measuring the difficulty preoperatively is important because it is correlated with the operative time and with increased complication rates.

To better predict the difficulty of surgery, Schneider et al. developed a “Thyroidectomy Difficulty Scale” (TDS), which has been recognized by a large number of scholars. The TDS was completed independently by two surgeons immediately after thyroidectomy [4]. Based on four items (vascularity, friability, mobility or fibrosis, and gland size), each item was scored on a scale from one to five, and the total score was 20 points. Similar to our findings, the authors concluded that the higher the TDS scores were, the longer the operative time and the higher the rates of complications. In this study, the patients with hyperthyroidism tended to score higher in vascularity, while those with Hashimoto’s thyroiditis tended to score higher in the mobility/fibrosis category. However, surgeons cannot use the TDS as a preoperative prediction tool, as the TDS can only be applied after surgery and has certain subjectivity.

To the best of our knowledge, the current research on the difficulty of thyroidectomy is very limited, and no study has quantified the difficulty of thyroidectomy preoperatively. Hence, more objective and efficient measures are needed.

This study used the operative time as an objective measure to define DT and found a higher postoperative complication rate in the DT group than in the NDT group. Multiple-factor logistic regression analyses confirmed that male sex, younger age, BMI, thyroid volume and TPO-Ab were independent preoperative predictors for DT. For the first time, we established and validated a nomogram for the preoperative evaluation of the difficulty of thyroidectomy. Using this nomogram, we can objectively evaluate the operative difficulty before thyroidectomy, which helps to perform individual risk assessment, predict postoperative complications and optimize the schedule of the operating room. Moreover, for DT, it is necessary to select very experienced surgeons in advance and to provide adequate physician–patient communication.

Previous studies have shown that hyperthyroidism, thyroiditis, male sex, age < 45 years, goiter and BMI are related to DT. In 2017, Kwak et al. showed that younger age and male sex were related to DT in terms of operating time [7]. Similarly, our study also found that younger age and male sex were predictive risk factors for DT. This may be due to the tough neck muscle tissue and dense interstitial space, which would require more time to dissect the flap and tissue. In our study, all patients received preoperative laryngoscopy; since only 2 patients were diagnosed with preoperative vocal cord paralysis, this factor was not assessed in this paper.

Hyperthyroidism is caused by an increase in the synthesis and secretion of thyroid hormones due to the hyperfunction of the thyroid gland, often accompanied by a decrease in TSH. In patients with hyperthyroidism, the thyroid gland is often characterized by hypervascularity and increased vascular fragility with abundant blood flow, which increase the difficulty of surgery. Schneider et al. and Vieni et al. discovered that patients with hyperthyroidism had higher scores on the vascularity item and believed that patients with hyperthyroidism were more difficult to perform surgery [4, 6]. Mok et al. also found that the diagnosis of hyperthyroidism was associated with more difficult thyroidectomy and more complications [5]. In contrast, the study of Frank et al. found that the operation time and blood loss of patients with hyperthyroidism were not significantly different from those of other patients [9]. Hence, they believed that hyperthyroidism does not increase the difficulty of thyroidectomy. Our study also did not find an association between free T3, free T4, TSH and DT, possibly because all patients with hyperthyroidism in our center had well-controlled thyroid function and iodine preparation was strictly carried out before surgery to maintain their basal metabolic rate within the normal range. All of the above are beneficial to reduce thyroid blood flow and intraoperative bleeding, avoid surgical complications, and thus reduce surgical difficulty.

Hashimoto thyroiditis (HT) is an autoimmune disease. In patients with HT, too much TG-AB and TPO-Ab are produced, destroying thyroid tissue and leading to fibrosis of the gland, which may increase the difficulty of thyroidectomy [10]. Multiple studies have suggested thyroiditis as a contributing factor to DT [5, 11, 12]. McManus et al. showed that the incidence of thyroidectomy complications in patients with HT was higher than that in those without HT, suggesting that thyroiditis leads to DT [11]. When analyzing the factors affecting the operative time of thyroidectomy, Kwak et al. found that the increase in TG-AB was related to the longer time of thyroid surgery [7]. Conversely, Consorti et al. believed that thyroiditis would not affect the time of thyroidectomy. In this study, TPO-Ab was an independent risk factor for DT [13]. However, TG-AB was associated with DT in the univariate analysis, but statistical significance was not observed in the multivariate analysis. The difference may be related to the insufficient sample size in our study.

Generally, enlarged nodules or glands are rich in blood supply, which will cause poor movement of the tumor, compress blood vessels and nerves, and cause limited surgical space, resulting in more difficult surgery [14]. Kwak et al. found no significant relationship between nodule size and DT [7]. Agarwal et al. found that the operation time and incidence of complications increased with the enlargement of the thyroid gland [15]. Vieni et al. also believed that goiter is a risk factor for DT, and a preoperative ultrasound measurement of the thyroid volume is necessary to assess the difficulty of thyroidectomy [6]. Consistent with previous studies, our research also showed that thyroid gland volume was positively associated with thyroidectomy difficulty, whereas nodule size was not.

Regarding the effect of BMI on DT, Milone et al. found that patients with BMI > 25 kg/ cm^2^ had longer operative time than those with normal BMI [16]. Buerba et al. found that BMI was positively correlated with the operative time and incidence of complications, suggesting that higher BMI was a predictor for DT [17]. The results of this study also support that BMI is an independent risk factor for DT, but some studies have suggested that neck circumference is associated with surgery difficulty, not BMI [13]. There is also a certain correlation between neck circumference and BMI. As a retrospective analysis, it is difficult to collect information on neck circumference. Further research is needed on the influence of the two on the difficulty of thyroidectomy.

There are some limitations to this study. First, this study is a retrospective single-center study with a limited sample size, and whether the risk factors and predictive nomogram for DT can be widely used requires external validation in further research. Second, this study did not explore the relationship between surgical difficulty and prognosis. Nevertheless, we will address these issues in future studies.

## Conclusion

In conclusion, this study found that DT was associated with higher complication rates and indicated that male sex, younger age, BMI, thyroid volume and TPO-Ab were independent preoperative predictors for DT. For the first time, we established a preoperative predictive nomogram that can objectively and individually assess the difficulty of surgery preoperatively, estimate the operation time and postoperative complications, help clinicians and nurses to better improve preoperative preparation, reasonably arrange the operating room time, and improve perioperative efficiency.

## Data Availability

The datasets used and/or analyzed during the current study are available from the corresponding author on reasonable request.

## Abbreviations

BMI: Body mass index
CI: Confidence interval
DCA: Decision curve analysis
DT: Difficult thyroidectomy
FT3: Free triiodothyronine
FT4: Free thyroxine
HT: Hashimoto thyroiditis
NDT: Nondifficult thyroidectomy
RLN: Recurrent laryngeal nerve
ROC: Receiver operating characteristics
TDS: Thyroidectomy Difficulty Scale
Tg-Ab: Thyroglobulin antibody
TPO-Ab: Thyroid peroxidase antibody
TSH: Thyroid-stimulating hormone
TV: Thyroid volume
